# Disordered eating behavior among group fitness instructors: a health-threatening secret?

**DOI:** 10.1186/s40337-015-0059-x

**Published:** 2015-06-24

**Authors:** Solfrid Bratland-Sanda, Merethe Pauline Nilsson, Jorunn Sundgot-Borgen

**Affiliations:** Department of sport and outdoor life sciences, Telemark University College, Gullbringvegen 36, 3800 Bø, Telemark Norway; Research Institute, Modum Bad Psychiatric Center, Vikersund, Norway; Department of sports medicine, Norwegian school of sport sciences, Oslo, Norway

**Keywords:** Sports, Exercise, Dieting, Body dissatisfaction, Psychiatry, Eating disorders

## Abstract

**Background:**

The present study aimed to examine disordered eating behavior (DE) and self-reported eating disorders (ED) among Norwegian group fitness instructors.

**Methods:**

Group fitness instructors from Norway (n = 685 females and 152 males, response rate: 57 %) completed an online survey. The survey included the instruments Eating Disorders Inventory (EDI) and the Exercise Dependence Scale (EDS).

**Results:**

A total of 22 % of the male and 59 % of the female respondents were classified with DE. The respondents classified with DE had higher BMI, more weight loss attempts, and higher total EDI score compared to the respondents with no DE. A correlation between EDI total score and EDS total score was found among both male and female group fitness instructors. No males and four percent of the females reported having a current ED. The instructors with self-reported current ED had higher weekly volume of instructing classes compared to the other instructors. None of the respondents with self-reported ED had informed their center manager about it.

**Conclusion:**

The high prevalence of DE behavior calls for concern. The reported secrecy regarding self-reported ED might decrease the possibility for early recognition and intervention. The findings reveal implications for the instructors’ physical and mental health, for their reputation and impact as important healthy role models and health/fitness authorities, and for the importance of prevention, identification and management of such behavior in fitness center settings.

## Background

The fitness center industry has shown a vast growth for the past two decades, and the number of group fitness instructors is continuously increasing worldwide. The industry shows massive focus upon body weight, shape and appearance, and classes called “fat burner”, “booty blast” and “body sculpting” are just a few examples of this emphasis. The group fitness instructors are considered important role models and health/fitness authorities for the fitness center attendees, both in the way they act and by their appearance [1]. Studies have shown that especially female exercise class participants prefer instructors that look fit and have a slim body type [2]. Furthermore, a study found aerobics instructors to focus greatly upon weight loss and calorie burning, instead of enjoyment in their communication with the participants at their classes [3].

Body dissatisfaction and dieting behavior are considered risk factors for the development of disordered eating behavior (DE) and eating disorders (ED) [4]. A high percentage of physically active women and athletes are reporting dieting and high degree of body dissatisfaction although they are not overweight [5-7], and these two factors have been shown to predict ED in ballet dancers [8]. Previous studies have found a prevalence of self-reported ED among group fitness instructors to vary from 5-40 % [9-13]. The majority of the instructors with self-reported ED in these studies reported remission from the disorder, although the scores on the Eating Disorders Inventory (EDI) were comparable to scores obtained from persons with anorexia nervosa [10]. All available studies have included female instructors only, and the sample size varies from 30 to about 370 instructors. Although some of these studies have included instructors teaching various types of classes, the focus has been upon aerobics instructors. The fitness industry have changed from mainly aerobics classes towards a wider range of classes such as strength training, endurance training without coordination, indoor cycling, and more body-mind related classes such as yoga and Pilates. Although the majority of instructors and attendants at group fitness training classes are women [14], it is important to acknowledge that DE and ED also occur among males [15]. The population of group fitness instructors is comparable to populations such as male and female dancers and leanness athletes. Higher prevalence of DE and ED among such athletes compared to non-leanness athletes [5], and dancers compared to non-dancers [16], makes it interesting to examine the DE prevalence among both male and female group fitness instructors. Furthermore, none of the previously published studies has included male group fitness instructors in their samples. Studies representing the general population, and from specific populations such as athletes and dancers, show higher prevalence rates of DE and ED among females compared to males. It is therefore of interest to examine possible gender differences in DE and ED among group fitness instructors.

A core symptom occurring throughout the whole spectrum from DE to ED is exercise, which is excessive in amount and/or compulsive in cognition [17, 18]. If the exercise volume increases beyond the positive health effects, it can eventually cause low energy availability and related health problems, dominate the entire existence, and reduce quality of life [19, 20]. Different cut offs for excessive exercise have been used. For individuals with DE, cut offs vary from ≥5-21 h/w of exercise, and that the behavior was compulsive, and out of control [21-23]. A strict cut off point for excessiveness of the exercise volume is difficult, as it depends on physical fitness, nutrition and hydration status, type of exercise performed and not at least the motive/reason for exercise [20]. In a Swedish study, 15 out of 18 aerobics instructors with ED had six or more hours of exercise per week [9]. This was considerably more compared to instructors with a previous ED or no reported ED. Unfortunately, this study did not separate between the amount of exercise performed through classes with clients, and exercising besides instructing. Furthermore, there is a lack of examination of the cognitions linked to the exercise behavior among the instructors. Previous studies have shown exercise to be both excessive in amount and compulsive in cognition among persons with ED [24, 23, 25]. Therefore, both these aspects of exercise behavior need to be examined in relation to DE among group fitness instructors.

Based on previous findings of high prevalence of ED among female aerobics instructors [10, 11, 9, 12, 13], and in comparable populations [5, 26, 27], the aim of this study was to examine the prevalence of DE and self-reported ED in male and female group fitness instructors in Norway. We hypothesized that instructors classified with DE exercised more per week, and scored higher on exercise dependency compared to instructors with no DE. Furthermore, we hypothesized that there was an association between DE and exercise dependence among the instructors.

## Methods

### Sample

Group fitness instructors from the three largest fitness companies in Norway (Sats, Elixia and Spenst) were invited to participate in this study. Inclusion criterion was teaching a minimum of one class per week during the spring semester of 2009. Exclusion criterion was inability to understand Norwegian language. Of the 1,473 instructors contacted, 78 instructors had invalid contact information. They were therefore unavailable for the study.

### Ethics, consent and permissions

The instructors received written information about the aim and completing of the study, and gave their written consent to participation. The study was approved by the regional committee for medical ethics in Southern Norway (REK Sør).

### Procedure

This study was conducted as a cross sectional study using self-report through the online survey system Questback (www.questback.com). The leaders of group training at the fitness centers provided the instructors’ e-mail addresses for the research group. We did not have permission to receive personal information such as names and postal addresses, therefore instructors with invalid e-mail addresses were unavailable for participation. The instructors were contacted by e-mail, and received up to two reminders to those who did not respond.

### Assessment

The questionnaire included questions about age, height, body weight, education, numbers and types of classes taught per week, exercise history, menstrual status, weight regulation, and self-reported ED. The questions about ED concerned whether the instructors had a current or previous ED, the duration of the disorder, if they received or had received treatment, and if they had told about their ED to the leaders and/or other colleagues at the fitness center. They were further asked to elaborate on why they had chosen openness or secrecy about their ED. Data on previous ED report are presented elsewhere [28]. In this study, we present data on respondents with a self-reported current ED.

Additionally, the Eating Disorders Inventory (EDI) [29] was used to assess DE symptoms. This is a validated 64-item inventory, which covers the aspects of drive for thinness (EDI-DT), body dissatisfaction (EDI-BD), bulimia, ineffectiveness, interoceptive awareness, maturity fears, interpersonal distrust and perfectionism. The items were ranged on a six-point Likert scale from “never” to “always”. Higher score on EDI indicates more symptoms of disordered eating. Cronbach’s alpha for the 64 items of EDI was .74 and .79 for the male and female instructors respectively. The subscales EDI-DT and EDI-BD have previously been classified as the only EDI subscales that predict ED [8], and Garner et al. [29] suggested cut off score 15 for EDI-DT and 14 for EDI-BD.

Exercise Dependence Scale (EDS) [30] was used to assess symptoms of exercise dependence. This 21 item scale is developed based on proposed criteria for exercise dependence [19, 31] and consists of the subscales tolerance, withdrawal, intention effect, lack of control, time, reduction in other activities, and continuance. The items are ranged on a six-point Likert scale from “never” to “always”. Higher score on EDS total and EDS subscales indicate more symptoms of exercise dependence. Cronbach’s alpha for the 21 items was .88 and .87 for the male and female instructors respectively.

DE behavior was defined as meeting one or more of the following criteria: BMI ≤18.5 kg/m^2^, EDI-DT score ≥15, EDI-BD score ≥14, self-reported ED, self-reported menstrual dysfunction (primary amenorrhea, secondary amenorrhea, oligomenorrhea or short luteal phase), and/or reporting ≥5 weight loss attempts. These criteria were developed from criteria used in previous studies among adolescent and adult elite athletes [26, 27]. Due to age differences between the sample in this study and our previous study on adolescent elite athletes [27], cut off for number of weight loss attempts was increased from three or more to five or more attempts.

### Statistics

The statistical software IBM SPSS 19 was used to analyze the data. Descriptive variables are shown in mean (standard deviation) and percentages. Chi square and independent *t* test was used to explore statistical differences between male and female respondents, and between instructors with and without DE behavior and self-reported ED. Fisher’s exact test was used for analyses of nominal data with n < 5 in one group. Correlations between EDI total score and EDS total score were calculated using Pearson’s correlation coefficient. To examine the effect size of the t-tests and the Pearson’s r, Cohen’s d was calculated. Significance level was set to .05.

## Results

A total of 837 group fitness instructors (n = 152 males) responded to the study, this gives a response rate of 57 %. The female instructors were younger and reported teaching on average more classes per week compared to the male instructors (Table 1).

**Table 1:** Descriptive data among female and male group fitness instructors

	Female (n = 685)	Male (n = 152)	*Differences*	*p-value*	Total (n = 837)
Age, years, Mean (SD)	32.8 (8.3)	37.9 (9.5)	t = 6.6	0.000***	33.7 (8.9)
BMI, kg/m^2^, Mean (SD)	22.3 (2.4)	25.5 (2.4)	t = 13.6	0.000***	23.0 (2.7)
General education level: higher, n (%)	500 (73)	109 (72)	*χ* ^2^ = 0.10	0.75	609 (73)
Exercise education level: higher, n (%)	344 (50)	60 (40)	*χ* ^2^ = 5.75	0.02*	404 (48)
Instructing classes, hours/week, Mean (SD)	3.2 (3.1)	1.5 (2.9)	t = 6.2	0.000***	2.9 (3.2)
Exercise besides instructing classes, hours/week, Mean (SD)	4.2 (2.7)	5.9 (3.4)	t = 6.7	0.000***	4.5 (2.9)

BMI: Body mass index. *p < 0.05. ***p < 0.001

### DE behavior

Twenty-two percent of the male respondents and 59 % of the female respondents were classified with DE behavior (Table 2). No males and 27 (4 %) females reported currently having ED (Table 2).

**Table 2:** Prevalence of Disordered Eating (DE) behavior symptoms among male and female group fitness instructors

	Male (n = 152)	Female (n = 685)		
	*n (%)*	*n (%)*	*χ* ^*2*^ *(1)*	*p-value*
Self-reported current eating disorder^a^	0 (0)	27 (4)	-	0.009
BMI ≤18.5 kg/m^2 a^	0 (0)	17 (3)	-	0.054
EDI-DT ≥15^a^	1 (0.7)	25 (4)	-	0.067
EDI-BD ≥14^a^	4 (3)	76 (11)	-	0.001**
Self-reported menstrual dysfunction	-	199 (30)	-	-
Weight loss attempts ≥5	32 (21)	204 (30)	4.7	0.03*
Total respondents classified with DE	34 (22)	371 (54)	65.7	0.000***

^a^Fisher’s exact test was used due to low n (n < 5). BMI: body mass index. EDI-DT: Eating disorders inventory – drive for thinness. EDI-BD: Eating disorders inventory – body dissatisfaction. *p < 0.05. **p < 0.01.***p < 0.001

Both male and female respondents with DE had higher BMI, more weight loss attempts, and higher EDI total score compared to male and female respondents without DE (Table 3). Differences in EDS total score were detected among the DE vs no DE female respondents; no such differences were detected among the male respondents. No differences regarding modality of classes instructed or exercise performed besides instruction were found. Neither were there differences with respect to previous sports participation, modality of sports and previous competition level.

**Table 3:** Characteristics of group fitness instructors with DE, No DE and self-reported ED^a^

	Males					Females									
	No DE (n = 114)	DE (n = 38)	t	p	ES	No DE (n = 271)	DE (n = 414)	t	p	ES	No ED (n = 654)	ED (n = 27)	t	p	ES
Age (years)	38.6 (10.1)	35.3 (6.5)	1.8	0.07	0.39	32.6 (7.6)	33.0 (8.9)	0.6	0.52	0.05	33.0 (8.3)	29.0 (7.7)	2.4	0.02*	0.50
BMI (kg/m^2^)	25.1 (2.7)	26.7 (2.4)	3.0	0.003**	0.63	22.1 (1.9)	22.8 (2.6)	3.6	0.000***	0.31	22.5 (2.4)	22.8 (2.6)	0.7	0.46	0.12
Exercise besides instructing (hours/week)	6.0 (3.4)	5.7 (2.8)	0.4	0.67	0.10	4.3 (2.8)	4.2 (2.6)	0.2	0.86	0.04	4.2 (2.7)	5.0 (3.1)	1.5	0.13	0.28
*Endurance training (hours/week)*	3.7 (3.0)	3.3 (2.4)	0.7	0.48	0.15	2.5 (1.9)	2.6 (1.9)	0.6	0.52	0.05	2.5 (1.9)	3.0 (2.2)	1.2	0.21	0.24
*Strength training (hours/week)*	2.3 (1.8)	2.4 (1.9)	0.3	0.74	0.05	1.8 (1.5)	1.6 (1.3)	1.2	0.22	0.14	2.0 (1.3)	2.4 (1.4)	1.2	0.23	0.30
Instruction classes (hours/week)	1.4 (2.9)	2.1 (2.8)	1.4	0.17	0.25	3.3 (2.9)	3.2 (3.3)	0.1	0.26	0.03	3.2 (3.0)	4.7 (5.4)	2.4	0.02*	0.34
*Aerobics, dance classes (hours/week)*	0.2 (0.7)	0.4 (0.9)	1.4	0.17	0.25	0.5 (1.0)	0.6 (1.0)	1.1	0.26	0.10	0.5 (1.0)	0.7 (1.7)	1.2	0.25	0.14
*Indoor cycling (hours/week)*	1.7 (1.3)	1.4 (1.6)	1.2	0.23	0.21	0.8 (1.1)	0.9 (1.2)	0.2	0.83	0.09	0.9 (1.1)	0.9 (1.2)	0.7	0.74	0
*Endurance classes (hours/week)*	0.4 (0.9)	0.7 (1.0)	1.3	0.37	0.32	1.1 (1.3)	0.9 (1.1)	2.2	0.01*	0.17	0.8 (1.2)	0.9 (1.3)	0.7	0.47	0.08
*Strength classes (hours/week)*	0.3 (0.7)	0.3 (0.8)	0.3	0.68	0	0.7 (1.0)	0.6 (0.8)	1.7	0.10	0.11	0.6 (0.9)	0.6 (0.8)	0.2	0.87	0
*Body/mind classes (hours/week)*	0.02 (0.1)	0.1 (0.4)	2.3	0.02*	0.27	0.4 (1.0)	0.4 (1.0)	0.4	0.72	0	0.4 (0.9)	1.0 (2.3)	3.4	0.001**	0.34
Instructor experience (years)	7.4 (7.1)	7.7 (4.8)	0.2	0.81	0.05	8.7 (6.3)	8.9 (7.0)	0.3	0.75	0.03	8.9 (6.7)	6.8 (5.1)	1.6	0.11	0.35
Weight loss attempts (numbers)	1.3 (1.4)	12.4 (9.6)	12.1	0.000***	1.62	1.7 (1.4)	8.0 (9.8)	10.9	0.000***	0.90	4.8 (7.3)	15.2 (14.9)	6.6	0.000***	0.89
Weight gain attempts (numbers)	1.6 (2.1)	2.1 (5.5)	0.5	0.60	0.12	0.4 (1.8)	0.3 (1.5)	0.4	0.69	0.06	0.3 (1.7)	0.1 (0.6)	0.7	0.50	0.16
*EDI*															
EDI total score	11.1 (9.7)	16.5 (13.3)	2.6	0.01*	0.46	10.5 (8.7)	21.0 (19.1)	9.0	0.000***	0.71	14.9 (14.5)	47.8 (20.3)	11.3	0.000***	1.87
Drive for thinness	1.0 (2.0)	2.2 (3.5)	2.6	0.01*	0.42	1.5 (2.5)	4.2 (5.2)	8.7	0.000***	0.66	2.6 (3.8)	12.8 (5.1)	13.3	0.000***	2.27
Body dissatisfaction	2.2 (2.8)	5.4 (5.9)	4.4	0.000***	0.69	2.6 (3.8)	6.7 (7.0)	9.5	0.000***	0.73	4.5 (5.7)	12.7 (8.2)	7.2	0.000***	1.16
Bulimia	0.3 (0.8)	0.5 (1.3)	1.6	0.11	0.19	0.1 (0.4)	0.8 (2.1)	6.1	0.000***	0.46	0.3 (1.0)	5.3 (4.1)	20.3	0.000***	1.67
Interoceptive awareness	0.7 (1.8)	0.6 (1.3)	0.2	0.85	0.06	0.6 (1.4)	1.5 (2.6)	5.2	0.000***	0.43	1.0 (2.0)	4.3 (3.6)	8.1	0.000***	1.13
Maturity fears	2.1 (2.5)	1.6 (2.3)	0.9	0.36	0.21	1.7 (2.0)	2.1 (2.8)	2.5	0.01*	0.16	1.9 (2.8)	2.9 (3.6)	2.1	0.004*	0.31
Ineffectiveness	0.5 (1.5)	0.9 (2.0)	1.3	0.20	0.23	0.4 (1.2)	1.0 (2.4)	4.0	0.000***	0.32	0.7 (1.9)	2.4 (2.9)	4.5	0.000***	0.69
Perfectionism	3.1 (3.2)	3.0 (3.0)	0.2	0.87	.03	2.8 (3.0)	3.4 (3.2)	2.8	0.006**	0.19	3.1 (3.1)	5.4 (4.1)	3.9	0.000***	0.63
Interpersonal distrust	1.3 (1.9)	2.2 (2.9)	2.2	0.03*	0.37	0.8 (1.6)	1.1 (2.1)	2.2	0.03*	0.16	0.9 (1.8)	2.0 (2.4)	3.0	0.003**	0.52
*EDS*															
EDS total score	56.6 (12.2)	58.5 (12.6)	0.8	0.41	0.15	54.9 (11.2)	57.7 (12.2)	3.0	0.003**	0.24	56.1 (11.6)	65.0 (13.7)	3.9	0.000***	0.70
Withdrawal	7.8 (2.6)	9.1 (2.4)	2.2*	0.03	0.52	8.2 (2.6)	8.5 (2.6)	1.2	0.50	0.12	8.3 (2.6)	9.7 (2.8)	2.8	0.005**	0.52
Tolerance	9.8 (2.8)	9.6 (2.5)	0.3	0.77	0.07	9.3 (2.8)	9.5 (2.7)	0.7	0.50	0.07	9.4 (2.7)	10.1 (3.4)	1.4	0.18	0.23
Lack of control	8.1 (2.8)	7.6 (3.4)	0.9	0.39	0.16	7.4 (2.7)	8.1 (3.0)	3.2	0.001**	0.25	7.7 (2.9)	9.2 (2.9)	2.6	0.01*	0.52
Continuance	6.7 (2.8)	7.4 (2.8)	1.2	0.23	0.25	6.7 (2.8)	7.4 (3.3)	2.9	0.003**	0.23	7.0 (3.1)	8.4 (3.6)	2.5	0.02*	0.42
Intention effect	7.5 (2.3)	7.4 (2.1)	0.1	0.90	0.05	7.0 (2.0)	7.3 (2.3)	1.6	0.11	0.14	7.2 (2.2)	7.9 (1.9)	1.7	0.09	0.34
Time	10.0 (3.3)	10.3 (3.2)	0.6	0.57	0.09	10.2 (8.9)	10.3 (3.3)	0.3	0.78	0.01	10.2 (3.1)	11.6 (3.7)	2.4	0.02*	0.41
Reduction in other activities	6.6 (2.1)	7.1 (2.2)	1.2	0.22	0.23	6.0 (1.9)	6.4 (2.1)	2.8	0.005**	0.2	6.1 (2.0)	8.0 (2.3)	4.8	0.000***	0.88

^a^ No male respondents reported current ED. This comparison was only made for the female respondents. ES: effect size (Cohen’s d). EDI: Eating Disorders Inventory. EDS: Exercise Dependence Scale. *p < 0.05. **p < 0.01. ***p < 0.001

Correlation analyses showed a positive correlation between total EDI and EDS score among male and female group fitness instructors classified with no DE, and among females with DE (Table 4).

**Table 4:** Pearson’s correlation between EDI and EDS scores among the group fitness instructors

	Males			Females		
	No DE	DE	Total	No DE	DE	Total
	EDS total score	EDS total score	EDS total score	EDS total score	EDS total score	EDS total score
EDI total score	r = .46***	r = .19	r = .38	r = .36***	r = .39***	r = .38***
Effect size	n = 113	n = 34	n = 147	n = 292	n = 354	n = 646
	d = 1.04	d = 0.39	d = 0.82	d = 0.77	d = 0.85	d = 0.82

EDI: Eating Disorders Inventory. EDS: Exercise Dependence Scale. ***p < 0.001

### Self-reported ED

Of the 27 instructors reporting current ED, mean (SD) duration of the ED was 10.5 (5.7) years. Twelve (44 %) reported that they had received treatment for the ED; four of them were currently undergoing outpatient treatment. Mean (SD) duration of treatment was 1.7 (0.8) years. The females with self-reported ED were younger and taught more classes per week compared to those with no self-reported ED (Table 3). There were no differences in weekly volume of instructing indoor cycling, dance/aerobics classes, strength training classes or endurance classes. A higher weekly volume of instructing body/mind classes such as yoga and Pilates was reported among females with self-reported ED compared to those with no self-reported ED (Table 3). Reported weekly volume of exercising besides instruction did not differ between females with and without self-reported ED.

None of the instructors with self-reported current ED had been open about the disorder to the manager/leader at their fitness center. The reasons for keeping it as a secret included fear of dismissal, shame and embarrassment, fear of being stigmatized, and a perception that this is no others business. One instructor wrote *“It’s something I will not tell them. It is none of their business, and they cannot solve my eating disorder”*. Another instructor wrote *“I’m ashamed, I don’t want them to feel sorry for me, and I’m afraid to lose my job”*. One instructor perceived the disorder as “no big deal”, and that she was fully in control of it: *“It’s a way of keeping control, and it’s not hazardous for me. I keep a normal weight and look healthy”*. The three instructors who had told it to one or more of their instructor colleagues reported perception of openness as a way of helping others in the same situation. They all emphasized that they told it to instructors they considered as close friends, and that they trusted these instructors not to tell other colleagues about it.

## Discussion and conclusions

The main finding of this study is the high prevalence of DE among both male and female group fitness instructors. The DE prevalence rates were similar to those found among female elite athletes [26]. The prevalence of self-reported ED was somewhat lower compared with previous studies examining group fitness instructors [9, 10], and elite athletes [5, 32]. The difference in ED prevalence can be explained by differences in measurements. The study by Torstveit et al. [32] included clinical interview by experienced interviewers and thereby a possibility to divide between athletes with and without a DSM-IV diagnosis of ED. Since our study was based on self-report, instructors who report having a current ED actually may not have met the strict previous DSM-IV criteria. On the other hand, instructors reporting no ED might have been classified with such a diagnosis according to the DSM-IV criteria if a structural clinical interview was performed. Furthermore, we used conservative cut offs for EDI-DT, EDI-BD and weight regulation attempts, which reduces the chance of false positive classification of DE behavior. Torstveit et al. [32] found that about nine percent of female elite athletes underreported DE and ED when comparing self-report to clinical interview. One of the main reasons for underreporting is the fear of exclusion from the team. There is a possibility for a high number of false negatives in our study as well, as the instructors might experience fear of losing their job. More so, it is a possibility that they undeliberately underreport DE behavior because they consider it a natural part of being a group fitness instructor.

Although differences in EDI total and subscale score between instructors with and without DE was detected, the scores are comparable to normal controls [33, 34], and much lower compared to data from ED patients [25, 33, 35] and female aerobics instructors [10]. The female aerobics instructors in Olson et al. [10] were younger, only instructing aerobics classes, and the low sample size makes it difficult to generalize the results to all group fitness instructors. The EDI total and subscale scores between Olson et al. [10] and our respondents with self-reported ED are however comparable with each other, and with data from ED patients. Although similar scores were found between Clausen et al. [33] and our sample, it must be noted that their sample was much younger than the sample in our study. This age difference might account for the differences in EDI subscale scores. We therefore argue that the normative data from the group fitness instructors’ population are different from normative data from the general young adult population. Group fitness instructors in general are possibly more satisfied with body weight and shape, and show less drive for thinness and bulimia symptoms, compared to general college students. A healthy relationship with one’s body and health is crucial when teaching exercise classes as the instructors are important role models and health/fitness authorities for the exercise class participants. It is therefore a call for concern that our results showed EDI-BD scores within pathological values among three percent of the male and 11 % of the female respondents.

The mean EDI scores among instructors with self-reported current ED is within pathological reference values [29], this support the assumption of actual psychopathology among these respondents. This pathology can also be supported by the higher numbers of weight loss attempts among instructors with current ED and among instructors with DE behavior. The EDI showed similar consistency in this sample compared to other studies [36]. The EDI scores being higher among instructors with DE compared to those without DE shows that the inventory discriminates between DE and no DE behavior [36]. A weakness with the EDI is the less reliability found among male respondents [34]. In our study, the internal consistency was somewhat lower among the male respondents; this reduced reliability can be due to the items chosen. Especially the EDI-BD subscale includes body parts that are traditionally women’s concern. Inclusion of dissatisfaction with body parts such as chest, shoulders and upper arm could have given higher scores among the male instructors.

The higher EDS score among female group fitness instructors with DE compared to those with no DE was expected due to previous studies comparing samples with and without DE or ED [18, 37], and due to excessive and compulsive exercise as a central symptom of both DE and ED. The total EDS scores among both respondents with and without DE were however surprisingly high compared to other clinical and non-clinical samples [18]. In contrast to previous studies [38], we detected no gender differences in the mean EDS score. This is probably due to higher EDS score among the females in this study compared to previous studies. Possible explanations for the diverse findings from previous studies are among others differences in sample and population (group fitness instructors / exercise professionals compared to regular exercisers, patients and females from the general population). The EDS is not validated for exercise professionals, and among group fitness instructors some statements/items can be false high due to the exercise as an occupation. The reliability of EDS among group fitness instructors is similar to what other studies have found [39], but it is possible that the EDS has poor sensitivity and specificity regarding identifying symptoms of exercise dependence among exercise professionals. Nevertheless, the positive correlation between EDI and EDS score found among both males and females indicates an association between psychopathology in DE and pathological exercise behavior. We believe that exercise behavior, as a symptom of DE and ED is especially important to emphasize among persons who have exercise as their occupation. It is possible that part of their work motivation is to legitimate extreme exercise behavior and/or avoid withdrawal symptoms. Such motives can be in conflict with the instructors’ role as healthy exercise authorities.

Female respondents with self-reported ED reported a higher weekly volume of class instruction compared to those without self-reported ED. Surprisingly, the difference was not in instructing classes with high metabolic loading, such as indoor cycling, aerobics or other endurance classes, but in mind/body classes such as yoga. Although the effect size of this difference was small, it was unexpected due to the nature of such classes. One might speculate whether this could be due to the experience of energy deficiency [20], and that instructors with self-reported ED choose more classes with low metabolic loading as compared to the female instructors without ED. No differences in weekly exercise volume or instruction volume was found between respondents with and without DE. This was in contrast to our hypothesis, and might be due to the high mean exercise volume, both instruction and exercise besides instruction, found in both groups.

All fitness instructors who reported current ED had kept the disorder secret from their manager at the fitness center. The reasons varied, including fear of dismissal/losing the job, beliefs of privacy, and/or inessentiality of the disorder. Shame, embarrassment and guilt are negative feelings that are often present among persons with ED [40]. This can be important factors for the privacy beliefs, and for the fear of losing the job as an instructor. The belittling of the disorder and it symptoms can also be explained by shame, embarrassment and guilt, or it can be explained by an actual denial of the symptom severity among the instructors. Interestingly, the instructors that considered openness to colleagues as a way of helping others struggling with disordered eating behavior, still kept the disorder as a secret from their managers. This illustrates the conflicts between the perceived advantages of openness, e.g. helping others in the same situation, and the perceived disadvantages of openness, e.g. fear of dismissal. The fear of judgment and dismissal calls for a need for openness and competence among managers of the fitness centers. The instructors also need information about legal and non-legal reasons for dismissal, and that they have the same protection and rights as employees as in other comparable occupations.

The strengths of this study include the large sample size, and use of the validated instruments EDI and EDS. The limitation is the use of self-report data, lack of diagnostic interview to classify ED diagnoses, use of cross-sectional design, and a response rate of 57 %. It was impossible for us to conduct drop out analysis to explore possible differences between responders and non-responders of this survey. The geographical distribution of the responders is in accordance with total population and number of fitness clubs in the various counties and cities in Norway. There is also a fair distribution of male and female responders, age distribution of the instructors, and responders instructing different types of classes. Thus, in spite of a response rate of 57 % we consider the sample as representative of the total population of group fitness instructors. On one hand, we cannot exclude the possibility that those who responded to this survey are more preoccupied with overall health and show more psychopathology regarding eating and exercise. On the other hand, it is possible that instructors with severe DE and ED refused to respond, leading to underestimated prevalence of both DE and current ED. We do acknowledge that our criteria for DE might underestimate such behavior among males, as the criteria relate more to typical female DE behavior. For instance, questions about weight loss attempts could have been changed to attempts to alter body weight in general, shape and/or fat/ mass/muscle mass. This could have given higher prevalence rates among the male respondents.

Implications of this study are the need for more awareness in the fitness center business regarding DE behavior among group fitness instructors. As the prevalence rates are higher than those of elite athletes in general [5, 32], sports physicians and sports physiotherapists need to be aware of the link between pathological exercise behavior and DE/ED among group fitness instructors. We suggest that when group fitness instructors seek treatment for exercise-related injuries, especially overuse injuries, they should be screened for DE and ED according to the recommendations for athletes [20]. The large numbers of instructors keeping their ED a secret is of concern. This is due to the detrimental health consequences of the ED, and also the fact that these instructors are being viewed as healthy role models [2]. Such secrets can therefore be a threat to both the instructors’ own health, and to the health of the participants at their classes. Clinically, instructors’ who struggle with DE and ED are probably more likely to maintain or increase such behavior due to the belonging to an environment, which emphasize health, fitness, exercise, nutrition and certain body ideals. This has many similarities to athletes struggling with DE and ED. Managers at fitness centers therefore need to be aware of signs and symptoms of DE and ED, and it is necessary that they are capable to identify and have a dialog with instructors that show such signs and symptoms. As previously mentioned, the group fitness instructors serve as role models and authorities on health and fitness. If they communicate unhealthy and/or extreme exercise, eating and dieting behavior to class attendants, especially the vulnerable attendants can be inspired to adopt such unhealthy and extreme behavior [3, 13]. The findings of this study are based on self-report. Future studies should perform clinical interviews to examine severity and diagnosis of the ED, and thus to control for possible response bias from the self-report data. Davis et al. [41] has suggested exercise as both a developing and a maintenance factor for ED. It is therefore interesting to further examine whether the occupation as a group fitness instructor is associated with risk, trigger or maintenance factor for DE and ED.
